# RAC Activity in Keloid Disease: Comparative Analysis of Fibroblasts from Margin of Keloid to its Surrounding Normal Skin

**Published:** 2008-04-08

**Authors:** Erik Witt, Angeliki Maliri, Duncan Angus McGrouther, Ardeshir Bayat

**Affiliations:** Plastic & Reconstructive Surgery Research, Manchester Interdisciplinary Biocentre, University of Manchester, Manchester M1 7DN, UK;; Department of Plastic and Reconstructive Surgery, South Manchester University Hospital Foundation Trust, Wythenshawe Hospital, Manchester M23 9LT, UK; and; Paterson Institute for Cancer Research, University of Manchester, Manchester M20 4BX, UK

## Abstract

**Background:** Keloids are characterized by excess collagen deposition within the dermis. Although the exact cause of the potentially overactive fibroblasts has yet to be elucidated, many etiological possibilities have been suggested. As fibroblasts originating from keloids appear to have an increased migration and proliferation rate, cell-signaling studies examining these factors may offer an opportunity to further our understanding of the pathogenesis of this disease. One of such cell-signaling messengers is the enzyme Ras-related C3 botulinum toxin substrate (RAC), which has never been investigated in keloid scars. **Objective:** This study explores the role of RAC activity in keloid disease. **Method:** Primary fibroblast cell lines were established from the margin of keloid (KF) scars as well as from the surrounding normal tissue (NF) from one anatomical site of the same patient. Migration and proliferation assays were performed, comparing matching NFs and KFs, and after cell lysis, RAC activity was assessed. **Results:** Comparing fibroblasts from 3 different patients, KFs migrated (*P* < .05) and proliferated (*P* < .05) faster than NFs. The activity levels of RAC were increased in KFs compared with NFs. **Conclusion:** KFs migrate and proliferate faster than NFs. RAC activity increases in KFs when compared with NFs. Inhibition of RAC could lead to a new therapeutic approach.

Keloid scars (KS) are benign fibroproliferative growths believed to result from an aberrant scarring process following trauma to the dermis. Trauma as minute as a vaccination injection is recognized as potentially triggering a KS. In contrast to hypertrophic scars, KSs extend beyond the margins of the wound.^2^–^3^ Keloid scars usually do not regress spontaneously and may potentially reach masses of nearly 2 kg.^4^ Keloid scars may not manifest themselves until several years after the initial injury. The scar is generally located on the shoulders, chest, upper back, neck, or earlobes. It may, nevertheless, occur at any other anatomical site. Patients often present with severe pain and pruritus. Their sheer size may lead to psychological distress and mechanical impairment of movement. Current options for treatment involve local corticosteroid injections, surgery, radiation, or pressure therapy. Independent of the method used, recurrence rate has been shown to be around 50% to 70%.^5^

The pruritus and discomfort is often maximal over the margin of the KS. Clinically, this area often appears inflamed, and it is believed that it is where the expansion into the normal surrounding tissue takes place. Indeed, the margin of a KS was previously described as the “proliferative” area.^6^ Furthermore, the overall gene activity was recently found to be increased in the margin of a KS (unpublished data, July 2007). Keloid scars are characterized by excess collagen accumulation and, as such, cell signaling of fibroblasts originating from the margin of the keloid may play a major role in the pathogenesis. Interestingly, fibroblasts from different anatomical sites are heterogeneous^7^ and display variable gene transcription depending on their location. Studies comparing fibroblast cell-signaling activities should, therefore, involve anatomically adjacent samples.

Ras-related C3 botulinum toxin substrate (RAC) is a guanosine triphosphate hydrolase (GTPase) that acts as a switch in the cell-signaling cascade.^8^ When a GTPase is bound to GDP, it is inactive. Binding of GTP triggers an alosteric change by which the physical shape and activity of the GTPase is altered and only then can the latter bind to and activate an effector. The rate of binding to GTP or GDP is controlled by GTPase-activating proteins (GAPs) and guanine nucleotide exchange factors (GEFs). Guanine nucleotide exchange factors facilitate the binding of GTP to the GTPase, hence activating it. Conversely, GAPs accelerate the hydrolysis of the bound GTP to form GDP, inactivating the GTPase.

The enzyme RAC has been shown to be involved in the control of the cell migration behavior^9^ and proliferation^10^ of fibroblasts. Nevertheless, no studies have investigated the activity levels of RAC in KFs. As the margin of a KS has been described as the area of high proliferation, it is of interest to investigate the activity of an enzyme controlling cell proliferation and migration in this location.

## METHODS

### Tissue samples

**Figure 1:**
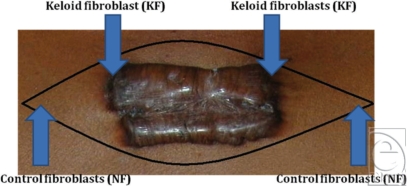
Schematic representation of the extralesional excision. Fibroblasts from the margin of the scar are entitled KF, and fibroblasts from the normal surrounding tissue, NF.

Patients that were to undergo the routine extralesional resection of KS were asked for consent to have the keloid tissue used for this project. The ethical approval was obtained from the local hospital and the research institute. Each scar was resected with a wide margin of unaffected tissue surrounding the scar. The unaffected tissue acted as control. After resection, the samples were divided into 2 different subgroups: tissue from the margin of the scar and from normal skin beyond the margin of the tumor. Figure 1 is a schematic representation of the extralesional excision. Fibroblasts obtained from the control tissue of the surrounding normal tissue are called normal fibroblasts (NF). The fibroblasts from the margin of the scar were entitled KF. The material was anonymized and transported to the Paterson Institute of Cancer Research. All scars were spreading beyond the margin of the initial wound and, as such, were clinically identified as KSs. All 3 samples were from patients of Afro-Caribbean descent. The first keloid tissue sample (KS1) was located on the scalp of a 36-year-old man. The second keloid sample (KS2) was located on the ear of a 33-year-old woman. The third sample (KS3) was resected from above the scapula of a 37-year-old woman. No previous surgery or adjuvant treatment had been performed on these scars.

### Cell culture

The samples were washed with phosphate-buffered saline (PBS) containing amphotericin solution B (Gibco, UK) and 1% penicillin/streptomycin (P/S) (Invitrogen, UK) mix for 15 minutes. The tissue was digested with 0.1% dispase (Becton Dickinson, UK) and neutralized with Dulbecco's Modified Eagle's Medium (DMEM) (Gibco, UK) plus 10% fetal bovine serum (FBS) (Gibco, UK) and 1% P/S. The epidermis and dermis were carefully separated using forceps. The dermis was diced and incubated with 0.5% collagenase type I (Lorne laboratories, UK). After filtration, the collagenase was neutralized and resuspended in 5 mL DMEM ± 10% FBS ± 1% P/S. The fibroblasts were grown in a 25-cm^2^ cell-culture flask (Cornig, UK) at 37°C and 5% CO_2_. The medium was changed every 3 days, and when the cells reached 90% to 100% confluence, they were passaged with 0.05% trypsin (Invitrogen, UK).

### Migration assay

The cell lines were plated on 6-well plates and grown to 100% confluent monolayer. When 100% confluence was reached, P1000 and P200 pipette tips were used to create a vertical and a horizontal scratch, respectively. Using a light microscope, pictures were taken from the same injury site at regular interval over the following 42 hours. The distance between the margins of the scratch was measured at constant magnification. The data was analyzed using the Student *t* test

### Proliferation assay

Approximately 2 × 10^4^ cells of each cell line were plated on 3 wells of 7 separate 6-well plates. Each 24 hours, one of these plates was fixed: The medium was washed off and well rinsed twice with PBS. The cells were then fixed with 4% formaldehyde in PBS for 15 minutes, followed by 2 washes with PBS and stored at 4°C in 2 mL of PBS with 0.1% sodium azide. When all wells were fixed, the plates were washed with PBS and stained with 0.2% crystal violet for 30 minutes. The fixed dry crystal violet of each well was dissolved, the absorbance of each sample was measured, and the data was analyzed using the Student *t* test.

### RAC activity assay

The cell lines were grown on 10-cm plates to about 80% confluence. The RAC activity assay protocol previously described by Sander et al^11^ was followed. The Cdc42/RAC interactive binding peptide (CRIB) used was prepared as indicated by Price et al.^12^ The cells were manually lysed and resuspended in the CRIB solution. Total RAC concentration was obtained by directly performing a Western blot on the supernatant from cell lysis. Passing the remainder of the supernatant through CRIB-specific Streptavidin-agarose (Sigma-Aldrich, UK) beads allowed the purification of the lysate by binding only GTP-bound RAC. The CRIB/GTP-bound RAC was resuspended, and the Western blot of this lysate represents the amount of active RAC (GTP-bound RAC).

### Western blot

Between 20 and 50 μg of protein was loaded into each lane. Nu-Page 4–12% Bis-Tris (Invitrogen, UK) gels were used for RAC assays. The gels were run under reducing conditions at 200 V for 25 minutes. The proteins from the gel were transferred onto a Millipore Immobilion-P polyvinylidene difluoride membrane (Billerica, MA, USA) at 30 V for 2~hours. After transfer, the membrane was blocked in 5% dry skimmed milk in Tween-PBS for 1 hour. Primary anti-RAC (BD Transduction Laboratories, USA) antibodies were used at 1:1000 dilution. After 1-hour incubation, the membrane was washed 3 times with Tween-PBS for 10 minutes. The secondary antimouse antibody (GE healthcare, USA) conjugated with horseradish peroxidase was used at 1:2500 dilution. Blots were visualized on chemiluminescence films by using Western Lightning Chemiluminescence Reagent Plus.

## RESULTS

### Migration

**Figure 2:**
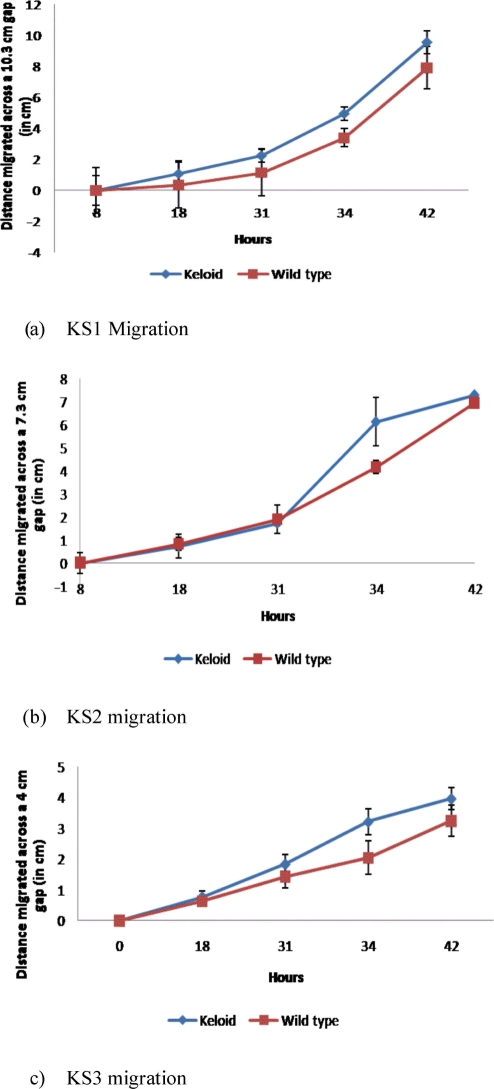
Fibroblast migration. The distance migrated across a gap of cells created by a pipette tip on a fully confluent cell culture. KS1, KS2, and KS3, respectively, show a significant higher migration rate after 34 hours in KFs than in NFs. After 42 hours, the difference in migration is nonsignificant, as the gap was crossed.

Fibroblast cell cultures were successfully established, and lines of passages 3 to 8 were used. Only cell lines originating from the same patient and having similar passages were compared. As the experiments were run in triplicates, experiments KS1 were performed with passages 4, 5, and 6; KS2 with passages 3, 4, and 5; and KS3 with passages 5,7, and 8. Figure 2 (a, b and c) shows the distance migrated by fibroblasts across the created gaps. The most significant difference in migration was observed after 34 hours when all 3 samples showed a significant increase in the migration of KFs when compared with NFs (*P* < .05). This measured increased migration rate is slowed after 34 hours as cells migrating from both edges of the gap reached each other.

### Proliferation

**Figure 3:**
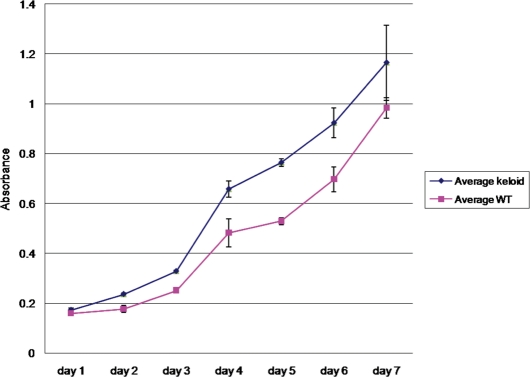
Absorbance of stained fibroblasts. Example of daily averages of absorbance readings (*n* = 3) of stained fixed-cell cultures. As the absorbance is directly related to the amount of cells in each well, the total number of cells present is proportional to the absorbance. Day 1 shows similar absorbance, proving that the amount of cells plated is similar between the 2 groups. On day 4, the proliferation is maximal and the KF rate of proliferation slows down compared with wild type (WT) because confluence is reached. Fibroblasts from the margin of the keloid proliferate at a faster rate than fibroblasts from the unaffected skin.

Proliferation rates represent the change in number of cells over a given period of time. By fixing and staining cells at equal intervals, the stain intensity is proportional to the amount of cells present. The absorbance-of-stained-cells measurements are reported in Figure 3. On day 1, *P* > .05, suggesting that the amount of cells plated are similar in both NF and KF samples. From day 2 to day 4, keloid absorbance increased at a much higher rate (*P* < .05). On day 4, the difference in growth was maximal. The difference in rate decreased between day 4 and day 7, and this is likely due to the wells reaching cell saturation. On day 7, near maximum confluence was reached, and the proliferation rate was not statistically different between the samples (*P* > .05). On day 4, KFs demonstrate a 36% higher proliferation than NFs (*P* < .05). This data confirmed that fibroblasts obtained from KSs proliferate at higher rates than those obtained from normal skin from the same patient.

### RAC activity

**Figure 4:**
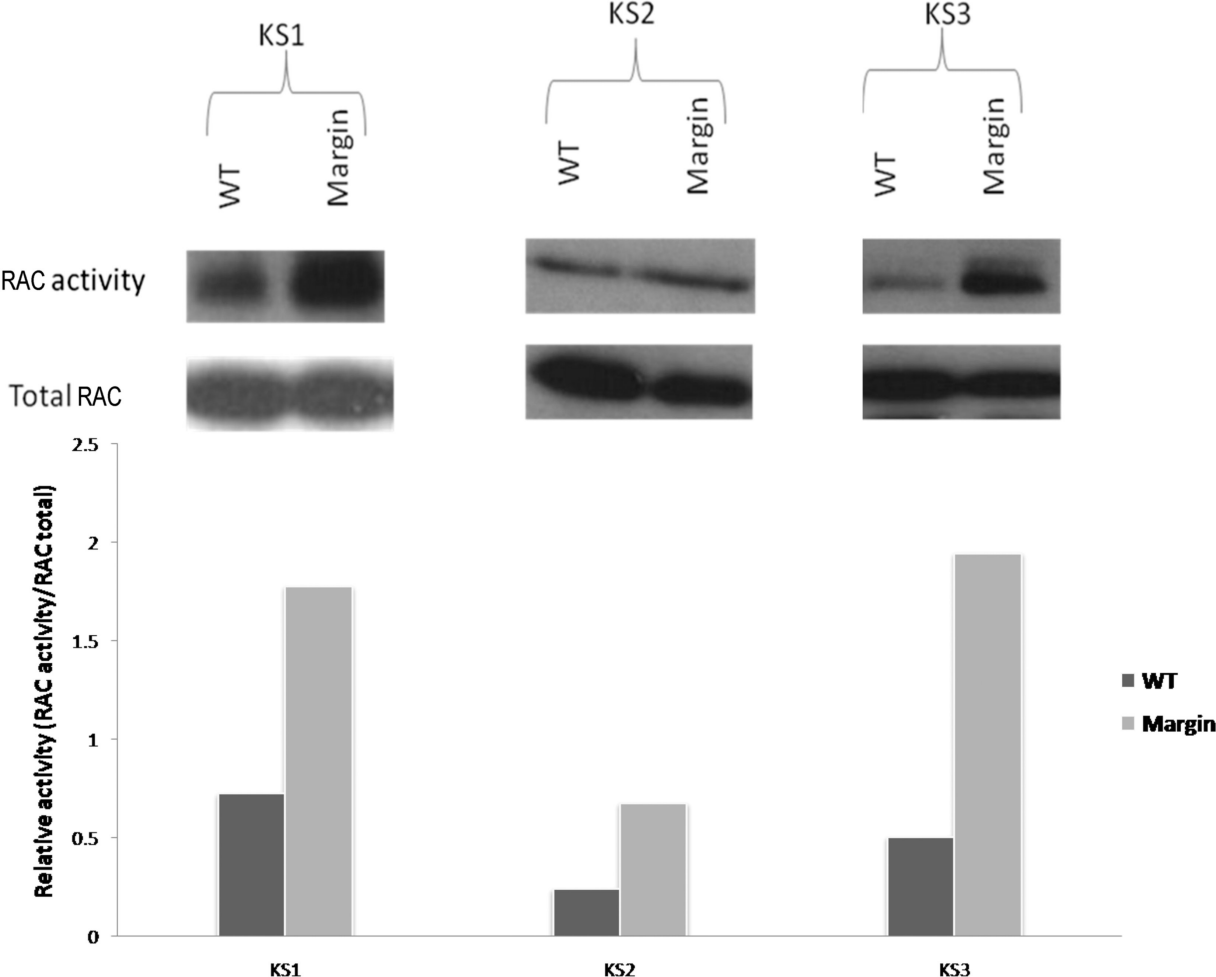
Ras-related C3 botulinum toxin substrate (RAC) activity Western blot. RAC activity of fibroblasts originating from normal tissue (WT) and from the margin of the keloid tissues (Margin) is depicted on these representative Western blots. Cell sample KS1 was located on the scalp of a 36-year-old man. KS2 was located on the ear of a 33-year-old woman. KS3 was resected from above the scapula of a 37-year-old woman. The Western blot bands were quantified using SynGene Gene Tools program. The intensity of each band was measured, and the ratio of RAC activity/RAC total was calculated to compare the relative activity in each sample. Fibroblasts originating from keloid margins show a higher RAC activity than fibroblasts from the normal surrounding skin (WT).

Figure 4 shows representative examples of results obtained from the Western blot for RAC activity and total concentration. A clear increase in RAC activity from the fibroblasts from the margin of the KS is observed. This finding of increased RAC activity has been reproduced in all assays. To better compare the activity between NF and KF, the SynGene Gene Tools program has been used to measure the intensity of each band. To best compare the activity of RAC between NF and KF, the ratio of active RAC to total RAC has been calculated. This shows that RAC activity is almost 3 times higher in KFs than in NFs.

## DISCUSSION

No previous studies have been published on RAC-activity levels in keloids. In this study, the assays showed an increase in RAC activity in KFs compared with that in NFs. By comparing samples from KSs and their matching control within similar passages, the authors believe that errors due to uncontrolled variability are minimized. The cell migration and proliferation assays show a clear difference between fibroblasts originating from KSs and the surrounding normal skin. Keloid fibroblasts proliferate and migrate faster than NFs. With *P* < .05, these results show strong evidence of a significant difference. Although results indicate a higher migration rate in KFs than that in NFs, it is important to note that migration assay results are often a combination of absolute cell migration and proliferation. The migration assays still provide strong data for a higher migration, as the difference of proliferation over a period of 34 hours is minimal. This interaction could nevertheless be reduced in future assays by using anti-proliferative agents (ie, mitomycin C).

The small sample size intrinsically creates uncertainties about statistical significance, although each sample was analyzed in a triplicate manner. Furthermore, observing an increased RAC activity across 3 different keloid samples is highly suggestive of a divergence. The enzyme RAC has been linked to several signaling cascades: as a downstream effector of the platelet-derived growth factor (PDGF) receptor as well as a downstream effector of the GTPase Ras. Therefore, any of these upstream factors could be a cause of an increase in RAC. The upstream factor overactivity could be due to intrinsic cell-signaling upregulation (constitutively active PDGF receptor), or due to an increase in external stimulus (eg, excess PDGF secretion by overlying keratinocytes). As previously described, GTPase activity is controlled by GAPs and GEFs. Upregulation or downregulation of GEFs would also lead to an increased activity of RAC. Whether this increase is a consequence of an intrinsic (fibroblasts) or an extrinsic (keratinocyte) trigger warrants future investigations.

This study showed a difference in migration and proliferation between KFs and NFs in the absence of overlying keloid keratinocytes (KKs). Previous observations^13^ indicate that KKs promote higher fibroblast migration and proliferation rates. Whereas this study does not contradict previous findings of KK interaction with KFs and NFs, it indicates that an underlying difference between NFs and KFs must exist in the absence of KKs. This observation would suggest that the initial trigger for keloid development could originate from within the fibroblasts. The RAC pathway could be investigated in matching keratinocytes, and in cocultures of fibroblasts and keratinocytes. It would be interesting to assess migration and proliferation in vitro in the presence of RAC inhibitors. Furthermore, activities of known GEFs and GAPs affecting RAC could be assessed to determine potential causes of the increased RAC activity.

Although the underlying molecular pathogenesis of keloid disease still remains unknown, this study shows for the first time that RAC activity is increased in fibroblasts originating from the margin of KSs. It can, therefore, be hypothesized that RAC is linked to the higher migration and proliferation of KFs. Which role this enzyme plays in the pathogenesis remains difficult to determine. Whether the increase in RAC activity is at the origin of the disease or whether it is a consequence of a different aberrant pathway is to be elucidated.

The margin of the KSs is a highly active area. This study reveals that the enzyme RAC has a higher activity in fibroblasts from the margin of the keloid when compared with the surrounding tissue. Furthermore, the fibroblasts from the margin migrated and proliferated faster than the normal tissue. The RAC has been shown to influence cell migration and proliferation rates, and as such, this study suggests that an increase in RAC activity in the margin of the KS might play a role in this proliferative wound. Further research into this field will yield a better understanding and potential management options for this benign, yet psychologically distressing, condition.

## ACKNOWLEDGMENTS

The authors thank Mr G White from the Paterson Institute for Cancer Research for the support and advice given.
